# The Physiology of the Iris

**Published:** 1858-01

**Authors:** 


*The Physiology of the Iris.*—A very interesting review of Professor Budge's work on this subject appears in the October number of the British and Foreign Medico-Chirurgical Review. The author shows the muscular fibres of the iris to be divisible into two distinct muscles, one radiating and acting as a *dilator*, the other circular, constituting a *constrictor*, of the pupil. The nerves of the iris are the sympathetic, the oculo-moter and the fifth pair. Of these, the sympathetic, when irritated, causes dilatation of the pupil; the oculo-moter under similar circumstances, induces contraction. Irritation of the optic nerve induces contraction, by reflex action through the corpora quadrigemina. Section of the fifth nerve brings about first contraction and then dilatation of the pupil, and after that sloughing of the cornea.

The centres of these different nerves have been established by direct experiment. That of the sympathetic, the *centrum cilio-spinale inferius*, as Budge calls it, is in the spinal marrow between the sixth cervical and the fourth dorsal vertebræ. The centre for contraction is to be found upon the inner surface of the corpora quadrigemina.

